# Impact of mineral fertilizers on mineral nutrients in the ginger rhizome and on soil enzymes activities and soil properties

**DOI:** 10.1016/j.sjbs.2021.05.037

**Published:** 2021-05-24

**Authors:** D. Jabborova, R.Z. Sayyed, A. Azimov, Z. Jabbarov, A. Matchanov, Y. Enakiev, Alaa Baazeem, Ayman EL Sabagh, Subhan Danish, Rahul Datta

**Affiliations:** a Laboratory of Medicinal Plants Genetics and Biotechnology, Institute of Genetics and Plants Experimental Biology, Academy of Sciences of Uzbekistan, Uzbekistan; b Department of Microbiology, PSGVP Mandal’s, Arts, Science & Commerce College, Shahada 425409, Maharashtra, India; c National University of Uzbekistan, Tashkent, Uzbekistan; d Institute of Bioorganic Chemistry of the Academy of Sciences of the Republic of Uzbekistan, Uzbekistan; e Agricultural Academy, “Nikola Pushkarov” Institute of Soil Science, Agrotechnology and Plant Protection, Sofia 1331, Bulgaria; f Department of Biology, College of Science, Taif University, P.O. Box 11099, Taif 21944, Saudi Arabia; g Department of Agronomy, Faculty of Agriculture, Kafrelsheikh University, Egypt; h Department of Soil Science, Faculty of Agricultural Sciences and Technology, Bahauddin Zakariya University, Multan, Pakistan; i Department of Geology and Pedology, Faculty of Forestry and Wood Technology, Mendel University in Brno, Zemedelska1, 61300 Brno, Czech Republic

**Keywords:** Ginger, Mineral fertilizers, Rhizome nutrients, Soil nutrients, Soil enzymes

## Abstract

Ginger is used as one of the important ingredients in traditional as well as modern medicine besides as a spice. It boosts immunity and is a rich source of many biologically active substances and minerals. Although it is a medicinally important crop, its productivity is, however, affected due to poor nutrient management and therefore it requires an adequate supply of nutrients in the form of inorganic fertilizers or organic manuring, or a mixture of both. In this context, the present study was aimed to investigate the effect of mineral fertilizers on the content of mineral elements in the ginger rhizome, on soil enzyme activity, and soil properties. Lysimeter experiments were conducted at the Institute of Genetics and Plant Experimental Biology, Kibray, Tashkent region, Uzbekistan. The experiment comprised of four treatments T1 – Control, T2 - N_75_P_50_K_50_ kg/ha, T3 - and T4 - N_100_P_75_K_75_ + B_3_Zn_6_Fe_6_ kg/ha. The results showed that the application of N_125_P_100_K_100_ kg/ha increased rhizome K content by 49%, P content by 20%, and Na content by 58% as compared to control without fertilizer. While the application of N_100_P_75_K_75_ + B_3_Zn_6_Fe_6_ kg/ha showed a significant enhancement in rhizome K, Ca, P, Mg, Na, Fe, Mn, Zn, Cu, Cr, Mo, and Si contents over the control. This treatment also improved active P content by 29%, total P content by 80%, total K content 16%, and N content by 33% content, and the activities of urease, invertase, and catalase activities as compared to control of without mineral fertilizer and control respectively. Thus the application of NPK + BZnFe at the rate of 100:75:75:3:6:6 kg/ha helps in improving macroelements and microelements in the ginger rhizome and activities of soil enzymes that helps in mineral nutrition of the rhizome.

## 1 Introduction

Medicinal plants are a major source of traditional as well as modern medicine and play a major role in the world (Egamberdieva and Jabborova, 2020, Jabborova et al., 2020a, Mamarasulov et al., 2020, Jabborova et al., 2021). Ginger (*Zingiber officinale* Rosc.) is a spice and medicinal plant belonging to the *Zingiberaceae* family. Ginger has long been used in folk medicine in India and China. Especially, the wet and dry root of ginger is widely used in the medicine and food industry (Jabborova and Egamberdieva, 2019). It has been used in folk medicine for colds, sore throats, asthma, and joint pain and stimulates appetite (Egamberdieva and Jabborova, 2018). Ginger is also rich in beneficial nutrients for example phosphorus, potassium, and calcium, which play important roles in human physiological processes. These substances play an important role in boosting human immunity and maintaining health (Jabborova et al., 2021, Zadeh and Kor, 2014). The dry rhizome of ginger is medicinal contains biologically active compounds. The rhizome contains carbohydrates, fats, proteins, vitamins, minerals, amino acids, monoterpenoids (camphene, sineiol, borneol, citral curcumin, and linalool), gingerol, and sesquiterpenoids.

The spice ginger is one of the most widely used species of the family *Zingiberaceae*. It is a common condiment for various foods and beverages (Jabborova et al., 2021). Both fresh and dried ginger rhizomes are used worldwide as a spice, and ginger extracts are used extensively in the food, beverage, and confectionery industries (Jabborova and Egamberdieva, 2019; Zingiber officinale, 2010). It is also chiefly used medicinally for indigestion, stomachache, malaria, fevers, common cold, and motion sickness. Besides being a key ingredient in many world cuisines and food processing industry, ginger possesses anti-carcinogenic, antioxidant, and anti-inflammatory properties (Zhao et al., 2016, Grzanna et al., 2005).

The production of this spice has been expanding in most parts of the world, as it can be grown under varied climatic conditions (Asfaw and Demissew, 2009). The productivity of ginger is, however, affected due to poor nutrient management Dinesh et al., 2012a), as it is a nutrient-exhaustive crop and therefore requires an adequate supply of nutrients at important stages of its growth (Weiss, 1997). Nutrient management options for this crop include inorganic or organic fertilizers or a mixture of both (Dinesh et al., 2012b). Effective nutrient management can help in reducing the overuse of chemical fertilizers, thereby safeguarding environmental quality. However, there are very few reports on the influence of different nutrient schedules on ginger yield and quality. Plant-derived foods have the potential to serve as dietary sources for all human-essential minerals (Minerals-Learn., 2010, Lokeshwari and Chandrappa, 2006).

The outcome of this study will ultimately help to ensure the dietary safety of society and improving both the quality and quantity of ginger. This study aimed to determine the levels of mineral nutrition in ginger, to assess the level of minerals in soil samples where the ginger was grown, and to correlate the levels of minerals in the ginger with that of soil in which it was cultivated.

## 2 Materials and methods

### 2.1 Experimental design

Ginger (*Zingiber officinale*) rhizome was used for lysimeter experiments. A lysimeter experiment was conducted to study the effect of mineral fertilizers on mineral nutrients of ginger and soil properties. The experiment was carried out in randomized block design with three replications a lysimeter experiments at the Institute of Genetics and Plant Experimental Biology, Kibray, Tashkent region, Uzbekistan. Experimental treatments included

T1 - Control
T2 - N_75_P_50_K_50_ kg/ha
T3 - N_125_P_100_K_100_ kg/ha
T4 - N_100_P_75_K_75_ + B_3_Zn_6_Fe_6_ kg/ha

Rhizomes were sown on 14 March for the year 2019. Harvesting was performed after 8 months of sowing.

### 2.2 Measurement of plant nutrients

Ginger rhizomes were harvested after 240 days of cultivation. Ginger rhizomes samples were prepared for analysis and were carried out in a special autoclave under the influence of hydrogen peroxide and nitric acid as disintegrating reagents for 6 h using a special microwave oven until the plant samples were converted into atomic elements. Sample volumes were accurately measured and 2% nitric acid (HNO_3_) was added. The analysis was carried out on an optical emission spectrometer with an inductively coupled argon plasma (2100DV (USA), (Sarabekov et al., 2021).

### 2.3 Analysis of soil nutrient and soil properties

Soil samples were collected from a lysimeter of the Institute of Genetics and Plant Experimental Biology, Kibray district, Tashkent province. To determination the soil properties before experimenting, soil samples took of soil. The mechanical components of the soil were determined by Kachinsky's method (Tursunov, 2010). Carbon and organic matter contents of soil were determined according to the method of Tyurin modified by CINAO (Soil, 2003). Mobile compounds of phosphorus and potassium were determined by the Machigin method modified by CINAO (Soil, 2005a). The total phosphorus and potassium contents were determined (Soils, 2005b). The total nitrogen content was determined according to the method of Soils (2002). The salinity level of soil was determined by water extraction methods (Pancu and Gautheyrou, 2006). Analysis of soil properties is shown in Table 1, Table 2, Table 3.

**Table 1:** The mechanical composition of irrigated soil in the Kibray district.

Land use types	Size of mechanical particle/mm							Physical mud	The mechanical content
	1–0,25	0,25–0,1	0,1–0,05	0,05–0,01	0,01–0,005	0,005–0,001	<0,001		
Cultivated land	4,35	6,89	10,99	36,18	12,64	14,99	13,96	41,59	Light sand

**Table 2:** The agrochemical properties of irrigated soil in the Kibray district.

Land use types	Active phosphorus and potassium, mg/kg		N-NO_3_, mg/kg	Total, %		N, %	Humus, %	C,%	C/N
	P_2_O_5_	К_2_O		P_2_O_5_	К_2_O				
Cultivated land	33.0	481.60	95.10	0.170	0.69	0.091	1.656	0.960	10,5

**Table 3:** The chemical properties of irrigated soil in the Kibray district.

Land use types	СО_2_ %	Alkalinity		Cl		SO_4_		Ca		Mg	
		Total НСО_3, %_	Total НСО_3,_ м.экв	%	mg/eq	%	mg/eq	%	mg/eq	%	mg/eq
Cultivated	5.41	0.023	0.08	0.056	0,20	0.080	0.50	0.230	11.48	0.07	5.73

### 2.4 Analysis of soil enzymes

The urease activity, and invertase, and catalase activity of soil were assayed according to the method of Guo et al. (2012) and Xaziev and Xaзиeв (2005), respectively. For the estimation of enzyme activities, a 2.5 g soil sample was added with 0.5 mL of toluene and incubated for 15 min. Then mixed and added to 2.5 mL of 10% urea and 5 mL citrate buffer in an incubator at 38 °C for 24 h. after incubation, it was filtered, then 4 mL of sodium phenate and 3 mL of sodium hypochlorite were added to 1 mL filtrate and diluted to 50 mL for 20 min. Enzyme activities were measured at 578 nm using a spectrophotometer. Urease activity was defined as the amount of enzyme that liberate NH4 per g of soil per h. Catalase activity was defined as the amount of enzyme that liberate oxygen per g soil while invertase activity was defined as mg of glucose liberated per g soil

### 2.5 Statistical analyses

All the experiments were performed in five replicates and the mean values of five replicates were considered. The data were statistically analyzed by one-way analysis of variance (ANOVA) and multiple comparisons of HSD employing the Tukey test with Stat View Software (SAS Institute, Cary, NC, USA). The significance of the effect of various treatments on plant growth parameters, plant nutrients, crop yield, and soil nutrients was determined by the magnitude of the *p*-value (*p* < 0.05 < 0.001).

## 3 Results

### 3.1 Rhizome nutrient contents

Ginger rhizome macroelements increased to NPK applications rate (125:100:100 kg/ha) and NPK + BZnFe applications rate (100:75:75:3:6:6 kg/ha). The NPK applications rate (125:100:100 kg/ha) increased significantly rhizome K content by 49%, P content by 20%, and Na content by 58% as compared to control no fertilizer. However, NPK + BZnFe applications rate (100:75:75:3:6:6 kg/ha) showed a significant enhancement in rhizome K, Ca, P, Mg, Na contents over the control (Table 4).

**Table 4:** The effect of mineral fertilizers on macroelements content of ginger rhizome.

Macroelements (mg/kg)	Treatments			
	Control	N_75_P_50_K_50_	N_125_P_100_K_100_	N_100_P_75_K_75_ + B_3_Zn_6_Fe_6_
K	10654.457	12243.321	15889.364*	20676.535*
Ca	2125.117	2414.534	10963.939*	20727.670*
P	3547.535	3791.722	4271.806*	5071.341*
Mg	4230.018	6185.985	8197.549*	8351.973*
Na	2323.383	3686.449	3479.408*	4250.860*

Data are means of three replicates (n = 3), * asterisk differed significantly at P < 0.05.

The ginger rhizome microelements were not significantly increased by control without fertilizer (Table 5). The low rate NPK (75:50:50 kg/ha) gradually increased rhizome Fe, Mn, Zn, Cu, Cr, Mo, and Si contents compared to control. Data regarding rhizome microelements content showed that NPK applications rate (125:100:100 kg/ha) significantly enhanced rhizome Fe, Mn, Zn, Cu, Cr, Mo, and Si contents over to control. The NPK applications rate (125:100:100 kg/ha) a significant rise rhizome Fe content by 26%, Mn content by 51%, Zn content by 41%, Cu content by 31%, and Si content by 71% compared to the control. A maximum number of rhizome micronutrient content was recorded with NPK + BZnFe applications rate (100:75:75:3:6:6 kg/ha) which resulted in rhizome Fe, Mn, Zn, Cu, Cr, Mo, and Si contents increase over the control and low rate NPK (75:50:50 kg/ha).

**Table 5:** The effect of mineral fertilizers on microelements content of ginger rhizome.

Microelements (mg/kg)	Treatments			
	Control	N_75_P_50_K_50_	N_125_P_100_K_100_	N_100_P_75_K_75_ + B_3_Zn_6_Fe_6_
Fe	178.717	196.228	225.672*	449.783*
Mn	78.266	80.944	118.724*	128.081*
Zn	2.277	2.808	3.166*	3.365*
Cu	1.317	1.720	1.727*	1.727*
Cr	0.608	0.733	1.116*	1.325*
Mo	0.123	0.143	0.249*	0.333*
Si	0.125	0.140	0.214*	0.305*

Data are means of three replicates (n = 3), * asterisk differed significantly at P < 0.05.

Data regarding the ginger rhizome ultramicroelements content showed that all treatments decreased rhizome Li, Be, V. Co, Ni, Ga, Ge, Ag, Cd, Sn, Sb, Cs, and Pb content (Table 6). The results showed that the ginger rhizome was not In, Ta, Re, Hg, and Tl.

**Table 6:** The effect of mineral fertilizers on ultramicroelements content of ginger rhizome.

Ultramicroelements (mg)	Treatments			
	Control	N_75_P_50_K_50_	N_125_P_100_K_100_	N_100_P_75_K_75_ + B_3_Zn_6_Fe_6_
Li	0.261	0.264	0.264	0.264
Be	0.007	0.009	0.013	0.012
V	0.234	0.302	0.777	0.624
Co	0.047	0.047	0.047	0.047
Ni	0.351	0.361	0.361	0.361
Ga	0.150	0.183	0.212	0.312
Ge	0.001	0.001	0.001	0.001
Nb	0.003	0.003	0.003	0.006
Ag	0.007	0.010	0.018	0.020
Cd	0.001	0.001	0.001	0.001
In	0.000	0.000	0.000	0.000
Sn	0.116	0.239	0.370	0.379
Sb	0.008	0.009	0.009	0.009
Cs	0.002	0.002	0.003	0.003
Ta	0.000	0.000	0.000	0.000
W	−0.001	−0.001	−0.001	−0.001
Re	0.000	0.000	0.000	0.000
Hg	−0.288	−0.287	−0.287	−0.287
Tl	−0.003	−0.003	−0.003	−0.003
Pb	0.092	0.093	0.065	0.065

Data are means of three replicates (n = 3), * asterisk differed significantly at P < 0.05.

### 3.2 Soil agrochemical and chemical properties

The results of soil mechanical composition are listed in Table 7. The data knotted that the increased fertilizers combinations of T2- N_75_P_50_K_50_ kg/ha, T3-N_125_P_100_K_100_ kg/ha, and T4 - N_100_P_75_K_75_ + B_3_Zn_6_Fe_6_ kg/ ha increased the mechanical composition of the soil (1–0.25 mm, 0.25–0.1 mm, 0.1–0.05). Whereas treatment 4 including macro and micronutrients N_100_P_75_K_75_ + B_3_Zn_6_Fe_6_ kg/ ha which had the highest amount of fertilizers was significantly increased soil mechanical particles (1–0.25 mm, 0.25–0.1 mm, 0.1–0.05) as compared to control.

**Table 7:** The effect of mineral fertilizers on the mechanical components of irrigated soil in the Kibray district.

Treatments	Factions, %							Physical mud (%)	Mechanical content
	1–0,25	0,25–0,1	0,1–0,05	0,05–0,01	0,01–0,005	0,005–0,001	<0,001		
Control	1.35	1.88	12.55	38.68	10.00	20.99	14.55	45.34	light sand
N_75_P_50_K_50_	2.05	2.00	15.40	36.53	9.20	19.60	15.22	44.02	light sand
N_125_P_100_K_100_	3.01	2.20	15.11	38.40	10.52	15.36	15,40	41.28	light sand
N_100_P_75_K_75_ + B_3_Zn_6_Fe_6_	4.78	3.14	15.00	36.77	9.70	16.51	14.10	40.31	light sand

Data are means of three replicates (n = 3), * asterisk differed significantly at P < 0.05.

The lowest level of total P content, total K content, N content, organic matter, active phosphorus, and potassium was evident in the soil without mineral fertilizer treatment (Table 8). The highest values of total P content, total K content, N content, organic matter, active phosphorus, and potassium were observed in soil with mineral fertilizer treatments NPK applications rate (125:100:100 kg/ha) and NPK + BZnFe applications rate (100:75:75:3:6:6 kg/ha). The NPK + BZnFe applications rate (100:75:75:3:6:6 kg/ha) enhanced nutrient contents of soil compared to all other treatments. However, NPK + BZnFe (100:75:75:3:6:6 kg/ha) treatment significantly increased active P content by 29%, total P content by 80%, total K content 16%, and N content by 33% compared to the control of without mineral fertilizer.

**Table 8:** The effect of mineral fertilizers on agrochemical properties of irrigated soil in Kibray district.

Treatments	Active phosphorus and potassium, mg/kg		N-NO_3_, mg/kg	Total, %		N, %	Humus%	C,%	C/N
	P_2_O_5_	К_2_O		P_2_O_5_	К_2_O				
Control	34.88	245.46	12.01	0.21	0.84	0.09	1.65	0.96	9.7
N_75_P_50_K_50_	41.0	248.10	25.10	0.22	0.85	0.09	1.66	0.96	9.7
N_125_P_100_K_100_	42.4	254.10	36.10	0.23	0.88	0.10	1.67	0.97	9.7
N_100_P_75_K_75_ + B_3_Zn_6_Fe_6_	45.0*	259.10	37.10*	0.38*	0.98*	0.12*	1.72	1.00	8.3

Data are means of three replicates (n = 3), * asterisk differed significantly at P < 0.05.

As a result of the experiments, there was a change in chlorine and sulfate ions in the amount of chlorine ion in the control were 0.22 mg/eq. In the soil (Table 9). However, NPK + BZnFe applications rate (100:75:75:3:6:6 kg/ha) was 0.45 mg / eq. The NPK + BZnFe (100:75:75:3:6:6 kg/ha) treatment affected improving the soil properties.

**Table 9:** The effect of mineral fertilizers on chemical properties of irrigated soil in the Kibray district.

Treatments	CO_2_ (%)	Alkalinity		Cl		SO_4_		Ca		Mg	
		Total HCO_3(%)_	Total HCO_3,_ mg/eq	%	mg/eq	%	mg/eq	%	mg/eq	%	mg/eq
Control	8.20	0.02	0.36	0.05	0,22	1.06	0,50	0.21	10.40	0.07	5.12
N_75_P_50_K_50_	7.98	0.01	0.35	0.04	0,22	1.02	0,48	0.18	9.45	0.06	4.88
N_125_P_100_K_100_	8.15	0.23	0.32	0.03	0,21	1.00	0,46	0.17	9.88	0.05	4.62
N_100_P_75_K_75_ + B_3_Zn_6_Fe_6_	8.54	0.21	0.30	0.03	0,19	1.10	0,45	0.16	9.87	0.04	4.52

Data are means of three replicates (n = 3), * asterisk differed significantly at P < 0.05.

**Fig. 1:**
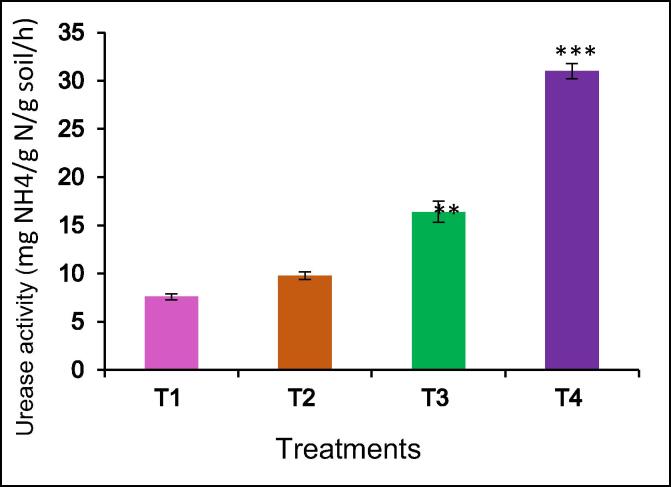
The effect of mineral fertilizers on urease activity of irrigated soil. Data are means of three replicates (n = 3), * asterisk differed significantly at P < 0.05*, P < 0.01**, P < 0.001***.

Data in Fig. 1 showed that mineral fertilizers increased the urease activity of soil. Urease activity increases reached a maximum at NPK applications rate (125:100:100 kg/ha) and NPK + BZnFe applications rate (100:75:75:3:6:6 kg/ha) treatments compared with the control. However, NPK + BZnFe applications rate (100:75:75:3:6:6 kg/ha) showed a significant increase in urease activity over the control.

### 3.3 Soil enzyme activity

**Fig. 2:**
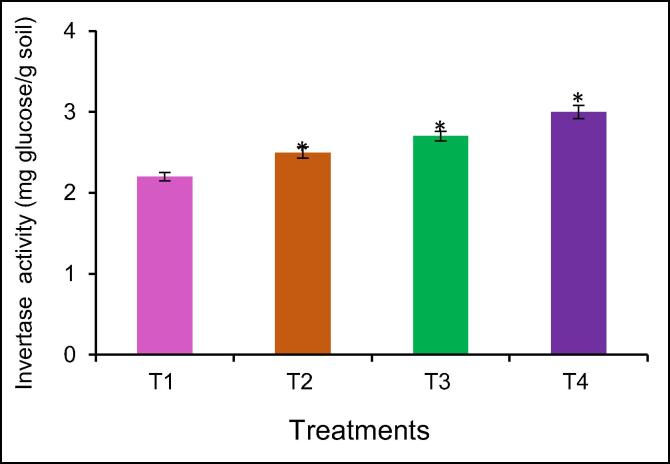
The effect of mineral fertilizers on invertase activity of irrigated soil**.** Data are means of three replicates (n = 3), * asterisk differed significantly at P < 0.05*, P < 0.01**, P < 0.001***.

The results showed that mineral fertilizers improved the invertase activity in the soil. The NPK applications rate (125:100:100 kg/ha) had significantly higher invertase activity by 23% than the control without fertilizer (Fig. 2). The maximum invertase activity was recorded in the NPK + BZnFe (100:75:75:3:6:6 kg/h) treatment. The NPK + BZnFe (100:75:75:3:6:6 kg/h) treatment increase significant invertase activity by 36% higher compared to control.

**Fig. 3:**
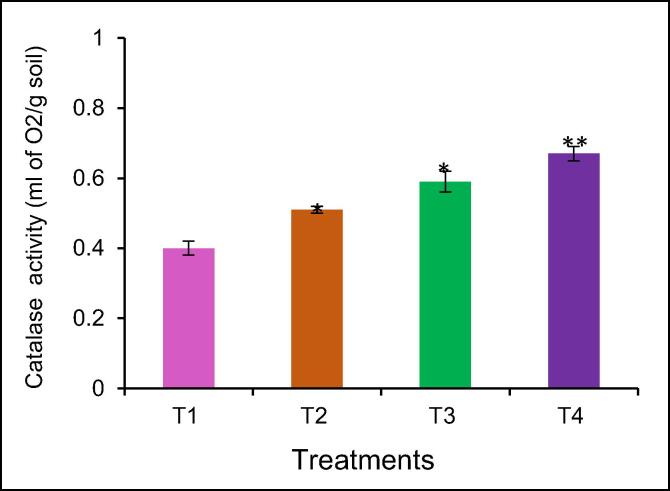
The effect of mineral fertilizers on catalase activity of irrigated soil district. Data are means of three replicates (n = 3), * asterisk differed significantly at P < 0.05*, P < 0.01**, P < 0.001*.

The data indicated that the increased fertilizers combinations of the NPK applications rate (75:50:50 kg/ha), NPK applications rate (125:100:100 kg/ha), and NPK + BZnFe applications rate (100:75:75:3:6:6 kg/ha) enhanced catalase activity in the soil (Fig. 3). The NPK applications rate (125:100:100 kg/ha) and NPK (125:100:100 kg/ha) treatments increased significantly the catalase activity by 27% and 47% compared to control without fertilizer. Combined macro and micronutrients NPK + BZnFe (100:75:75:3:6:6 kg/ha) significantly enhanced the urease activity by 67% compared to control.

## 4 Discussion

### 4.1 Impact of mineral fertilizers on rhizome nutrients of ginger

Mineral elements are important for human, animal, and plant nutrition. Several scientists have been studied macro–micro elements in plants (Egamberdieva et al., 2016, Egamberdieva et al., 2017, Egamberdieva et al., 2018, Sarabekov et al., 2021). N, P, K are an important role play plant growth, development, and yield productivity (Jabborova et al., 2018, Egamberdieva et al., 2018). The studied ginger rhizomes in this study are a source of macroelements, microelements, and ultramicroelements cultivating in the Tashkent Region, Uzbekistan. For the first time the study of the content of macro-elements and microelements of ginger in the soil climatic conditions of the Tashkent region, Uzbekistan, revealed that N, P, K, Ca, Mg, Na, Mn, Fe, Zn, and Cu are high amount in the rhizome (Tables 4, 5). Many studies have been conducted on analyzing the essential and non-essential metal content of ginger in Nigeria (Obiajunwa et al., 2002, Ogunwandea and Olawore, 2004, Aiwonegbe and Ikhuoria, 2007), India (Devi et al., 2008), Saudi Arabia (Al-Eed et al., 2002, Alwakeel, 2008) and Ethiopia (Wagesho and Chandravanshi, 2015). Olubunmi et al. (2013) reported that nutrient analysis of ginger indicated their richness in calcium, magnesium, sodium, potassium, phosphorous, manganese, iron, zinc, and copper. The literature showed that there are no studies on the determination of mineral nutrients in ginger cultivated in Uzbekistan. N, P, and K fertilizers play an important role in plant growth, plant nutrition, and yield. The study showed that The NPK applications rate (125:100:100 kg/ha) enhanced significantly rhizome K content by 49%, P content by 20%, and Na content by 58% as compared to control. Macro and micronutrient fertilizer NPK + BZnFe applications rate (100:75:75:3:6:6 kg/ha) showed a significant increase in ginger rhizome K, Ca, P, Mg, Na contents over the control (Table 4). Similar findings corroborating increased ginger nutrients such as N content, P content, and K content by the NPK applications rate (100: 60: 60 kg/h) were reported by Yanthan et al. (2010). A positive effect of mineral fertilizers on the uptake of nutrients by ginger was observed by Thakur and Sharma (1997). Singh and Singh (2007) reported that enhanced uptake of nutrients in ginger by inorganic fertilizers.

### 4.2 Impact of mineral fertilizers on agrochemical and chemical properties of soil

Soil nutrients are an important role play for plant growth and yield. Many authors reported that the nutrient contents in soil were analyzed plant cultivating before and after (Jabborova et al., 2019, Jabborova et al., 2020b). In the present study, we used mineral fertilizers application different levels to improve soil agrochemical properties of soil (Table 7). Mineral fertilizer treatments NPK applications rate (125:100:100 kg/ha) and NPK + BZnFe applications rate (100:75:75:3:6:6 kg/ha) was found to increase the soil agrochemical properties such as total P content, total K content, N content, organic matter, active phosphorus, and active potassium compared to the control treatment (Table 7). The impact of inorganic fertilizers on agrochemical and chemical properties in soil was observed by several researchers (McDowell et al., 2004, Fang et al., 2009, Monaco et al., 2008, Wang et al., 2008). This finding is consistent with the report of Dinesh et al., 2012a, Dinesh et al., 2012b who observed chemical nutrient management as a positive enhance total N of soil under rainfed ginger (*Zingiber officinale* Rosc.).Similar findings confirming increased the N content, P content and K content in soil by the NPK applications rate (100: 60: 60 kg/h) was reported by Yanthan et al. (2010). A recent study from Srinivasan et al. (2019) indicated that high mineral fertilizer decreased the N, P, K, Ca, Mg, and Fe content in the soil.

### 4.3 Impact of mineral fertilizers on soil enzymes

For the first time study of soil enzyme activity in soil cultivation ginger in Uzbekistan. Many studies have been conducted to determine soil enzyme activity in soil under cultivation ginger in India (Yanthan et al., 2010, Dinesh et al., 2012a, Dinesh et al., 2012b, Srinivasan et al., 2019). The data showed that the NPK + BZnFe applications rate (100:75:75:3:6:6 kg/ha) significantly increased urase (Fig. 1), invertase (Fig. 2), and catalase activity (Fig. 3) in soil. Similar findings confirming the NPK applications rate (75: 50: 50 kg/h) increased the urease activity by 27.0% in the soil as was reported by Srinivasan et al. (2019). This finding confirms earlier studies by Singh, 2015, Allison et al., 2007 both observed urease enzyme activity in soil by mineral fertilizers application. The NPK application rate (75:50:50 kg/ha) decrease urease activity compared to T-3 and T-4. Dinesh et al., 2012a, Dinesh et al., 2012b reported that the NPK application rate (75: 50: 50 kg/h) decreases urease activity. The literature showed that there are no studies on the determination of soil enzyme activity in cultivated ginger in Uzbekistan.

## 5 Conclusion

For the first time in Uzbekistan was studied the content of mineral elements of ginger rhizome cultivating in Uzbekistan. The NPK + BZnFe applications rate (100:75:75:3:6:6 kg/ha) increased significantly rhizome macro and micronutrients N, P, K, Ca, Mg, Na, Mn, Fe, Zn and Cu contents. A higher rate of NPK + BZnFe applications rate (100:75:75:3:6:6 kg/ha) mostly increased soil agrochemical properties total P content, total K content, N content, organic matter, active phosphorus, and active potassium compared to the control and other treatments. The highest activity of urease, invertase, and catalase in soil by the NPK + BZnFe (100:75:75:3:6:6 kg/ha) fertilization rate was observed. The combined application of the NPK + BZnFe (100:75:75:3:6:6 kg/ha) is a better source of nutrient input for obtaining higher ginger yield as well as in sustaining soil fertility under the Uzbekistan soil-climate conditions.

## Funding

This work was supported by the Ministry of Innovational Development of the Republic of Uzbekistan [project number A-ФA-2019-33, 2019–2022].

## Declaration of Competing Interest

The authors declare that they have no known competing financial interests or personal relationships that could have appeared to influence the work reported in this paper.
